# The role of deep convection and nocturnal low-level jets for dust emission in summertime West Africa: Estimates from convection-permitting simulations

**DOI:** 10.1002/jgrd.50402

**Published:** 2013-05-29

**Authors:** B Heinold, P Knippertz, JH Marsham, S Fiedler, NS Dixon, K Schepanski, B Laurent, I Tegen

**Affiliations:** 1 School of Earth and Environment, University of LeedsLeeds, UK; 2 Now at Leibniz Institute for Tropospheric ResearchLeipzig, Germany; 3 Laboratoire Interuniversitaire des Systèmes Atmosphériques, Laboratoire mixte Paris VII-UPEC-CNRSCréteil, France; 4 Leibniz Institute for Tropospheric ResearchLeipzig, Germany

## Abstract

[1] Convective cold pools and the breakdown of nocturnal low-level jets (NLLJs) are key meteorological drivers of dust emission over summertime West Africa, the world’s largest dust source. This study is the first to quantify their relative contributions and physical interrelations using objective detection algorithms and an off-line dust emission model applied to convection-permitting simulations from the Met Office Unified Model. The study period covers 25 July to 02 September 2006. All estimates may therefore vary on an interannual basis. The main conclusions are as follows: (a) approximately 40% of the dust emissions are from NLLJs, 40% from cold pools, and 20% from unidentified processes (dry convection, land-sea and mountain circulations); (b) more than half of the cold-pool emissions are linked to a newly identified mechanism where aged cold pools form a jet above the nocturnal stable layer; (c) 50% of the dust emissions occur from 1500 to 0200 LT with a minimum around sunrise and after midday, and 60% of the morning-to-noon emissions occur under clear skies, but only 10% of the afternoon-to-nighttime emissions, suggesting large biases in satellite retrievals; (d) considering precipitation and soil moisture effects, cold-pool emissions are reduced by 15%; and (e) models with parameterized convection show substantially less cold-pool emissions but have larger NLLJ contributions. The results are much more sensitive to whether convection is parameterized or explicit than to the choice of the land-surface characterization, which generally is a large source of uncertainty. This study demonstrates the need of realistically representing moist convection and stable nighttime conditions for dust modeling.

**Citation:** Heinold, B., P. Knippertz, J. H. Marsham, S. Fiedler, N. S. Dixon, K. Schepanski, B. Laurent, and I. Tegen (2013), The role of deep convection and nocturnal low-level jets for dust emission in summertime West Africa: Estimates from convection-permitting simulations, *J. Geophys. Res. Atmos., 118*, 4385–4400, doi:10.1002/jgrd.50402.

## 1. Introduction

[2] Some of the most important sources of airborne mineral dust are situated in West Africa, which comprises major parts of the Saharan desert and Sahel, contributing up to 50% to the global dust emissions *Luo et al*., 2003; *Washington et al*., 2003. As the largest fraction of particulate aerosol mass, mineral dust is an important player in the Earth system *Carslaw et al*., 2010; *Shao et al*., 2011. Mineral dust also has an impact on air quality and visibility with potentially adverse effects on human health, transportation, and the growing solar energy industry *Griffin*, 2007; *Breitkreuz et al*., 2007. The growing awareness of the importance of mineral dust is reflected by the increasing number of air quality and weather forecast models that consider transport and radiative effects of desert dust for answering scientific questions and improving the numerical weather prediction [e.g., *Tompkins et al*., 2005; *Perez et al*., 2006; *Balkanski et al*., 2007; *Rodwell and Jung*, 2008. General circulation models used for climate change research for long have included the atmospheric dust cycle, but increasingly interactions with the different cycles of the Earth system are accounted for in the simulations *Textor et al*., 2006; *Zhang et al*., 2012.

[3] Dust uplift occurs in dry, sparsely, or non-vegetated regions, when the wind shear stress on the surface exceeds a threshold that depends on land-surface properties such as soil texture and moisture, the degree of surface crusting, and the presence of roughness elements. The actual dust emission flux, i.e., the amount of emitted dust particle mass per area and time, is a function of the third or fourth power of the surface wind speed [e.g., *Bagnold*, 1941; *Marticorena and Bergametti*, 1995; *Shao et al*., 2011. This highly nonlinear relationship gives particular importance to meteorological processes that generate peak surface winds. Therefore, predicting dust emission events requires both detailed information on soil characteristics and an adequate representation of the upper range of the wind speed distribution in the model *Grini et al*., 2005.

[4] Over summertime West Africa, two main peak-wind-generating meteorological phenomena have been identified by observations from satellites and field campaigns in recent years: the nocturnal low-level jet (NLLJ) and the cold outflow from deep moist convection *Knippertz and Todd*, 2012. The breakdown of the nocturnal LLJ, which results from the downward mixing of momentum during the morning transition of the boundary layer, is associated with characteristic peak surface winds from morning to midday. A prominent example is the Bodélé LLJ *Washington and Todd*, 2005, but recently NLLJs have been shown to be important drivers of dust emissions across large parts of the Sahara [e.g., *Knippertz*, 2008; *Schepanski et al*., 2009; *Fiedler et al*., 2013. Strong dust-emitting winds also occur at the leading edge of the cold-pool outflow generated by evaporating precipitation from convective systems *Knippertz et al*., 2007; *Marsham et al*., 2008; *Williams et al*., 2009. The scale of these events ranges from microbursts to dust storms spanning several hundred kilometers, the so-called haboobs, which are caused by organized moist convection.

[5] African Easterly Waves (AEWs) and the large pressure gradient associated with the Saharan heat low (SHL) are primary controls of dust uplift over summertime West Africa on the synoptic scale *Knippertz and Todd*, 2010. Strong surface winds are either directly related to intense AEW surface vortices and accelerations at the leading edge of the monsoon inflow or indirectly by fostering the formation of NLLJs and convective cold pools. In addition, downscale dry convective plumes and dust devils make a yet unknown contribution to Saharan dust emissions [e.g., *Koch and Renno*, 2005; *Ansmann et al*., 2009.

[6] The relative contribution of these processes to dust emissions is still much debated. Uncertainties exist due to the fact that dust source observations by satellites are hampered by clouds or high contents of column water vapor *Brindley et al*., 2012; *Schepanski et al*., 2012. State-of-the-art atmospheric models struggle to represent the complex meteorological conditions in West Africa *Xue et al*., 2010. This is particularly true for moist convection, which is a subgrid-scale process for all global and most regional operational models and therefore needs to be parameterized. In many cases, a mass flux scheme is used, which estimates the vertical exchange of heat, moisture, and momentum within a single model column, and naturally fails to reproduce the structure and intensity of downdrafts, density currents, and propagating convective systems *Davis et al*., 2003. It is now possible to perform multiday runs with regional models at sufficiently high resolution (<5 km) to allow an explicit description of convection. Examples show that those simulations can much better represent the characteristics of deep convection and the behavior of spreading cold pools *Knippertz et al*., 2009; *Reinfried et al*., 2009; *Pearson et al*., 2010; *Barthe et al*., 2011.

[7] *Marsham et al*. 2011a, hereafter M11] used 10 day continental-scale convection-permitting simulations of summertime West Africa, produced in the framework of the *Cascade* project (http://climate.ncas.ac.uk/Cascade), to estimate the relative importance of haboobs and NLLJs in generating peak surface wind speeds and how this depends on the representation of moist convection. M11 did not model emission of mineral dust explicitly, but used a diagnostic parameter “uplift potential,” the wind speed-dependent component of a standard dust emission parameterization. Using the area-mean diurnal cycle as a quantitative indicator, M11 suggest that cold-pool outflows potentially contribute about half of the dust emissions. While haboobs are very poorly modeled using parameterized deep convection, the model tends to compensate the missing convective dust events by NLLJ-related dust uplift that result from a stronger SHL. This implies a shift in the diurnal cycle of dust emissions from the afternoon and evening to morning hours, and an underestimation of dust release in the Sahel.

[8] The present study expands the work of M11 in three aspects by

1. [9] using newly available 40 day convection-permitting model runs from the *Cascade* project for summer 2006,
2. [10] explicitly calculating dust emission fluxes with a sophisticated off-line model, and
3. [11] an automated identification of NLLJ and cold-pool related dust emission.

[12] The investigation tackles several scientific problems: (1) the relative contribution of NLLJs and cold pools to the West African dust production in summer; (2) possible links between the occurrence of cold pools and NLLJ formation; (3) the fraction of dust emission under clouds, which provides an estimate for potential biases in previous satellite-based studies; (4) the influence of soil moisture and instantaneous wash out, which answers the long-standing question of whether convective cold pools are important for Saharan dust emission despite the considerable amount of precipitation from their parent convective storms *Takemi*, 2005; *Tulet et al*., 2010; *Seigel and van den Heever*, 2012; (5) the influence of different data sets of prescribed soil surface properties; and (6) the sensitivity of the modeled dust emissions to the spatial resolution of the meteorological model and the representation of moist convection similar to M11.

[13] The paper is organized as follows. The meteorological model setup, the dust emission model, and the detection algorithms are described in section 2. Section 3 contains a detailed analysis of the diurnal cycle of modeled dust emission, followed by sensitivity studies on soil moisture, land-surface characterization, and model spatial resolution in section 4. In section 5, the case of an aging cold pool an its links to NLLJ formation are examined followed by a more general analysis of the importance of NLLJs and cold pools for the dust production. A summary and conclusions are provided in section 6.

## 2. Method

### 2.1. The *Cascade* Project Simulations

**Figure 1:**
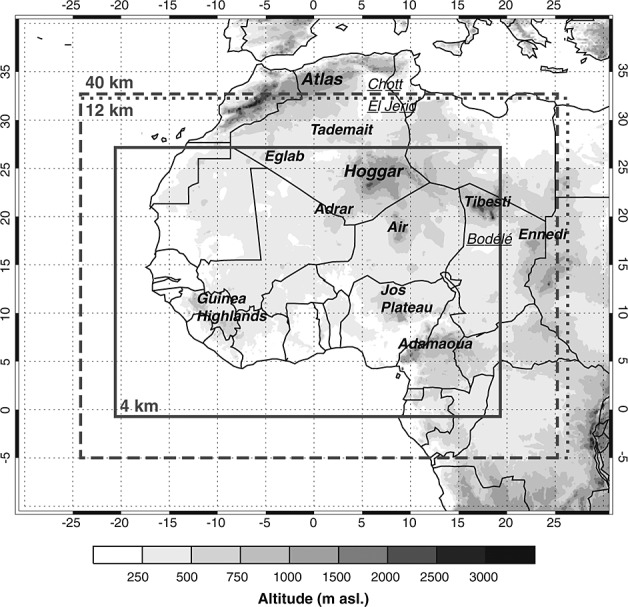
Model domains of Unified Model simulations performed within the *Cascade* project and geographical terms used in the text. Nested 40 km (dashed line), 12 km (dotted line), and 4 km (solid line) domains are shown.

[14] The simulations used in this study were performed with the UK Met Office Unified Model (UM; version 7.1) within the framework of the *Cascade* project. The study period spans the 40 days from 25 July to 02 September 2006, and the model domain covers West Africa including important parts of the Sahara desert (Figure 1). Using a one-way nesting technique, the model was run with 40, 12, and 4 km horizontal grid spacings. Operational analyses from the European Centre for Medium-Range Weather Forecasts (ECMWF) were used to initialize and force boundaries of the 40 km and 12 km runs, while the 4 km nest was driven with the initial and boundary conditions provided by the 12 km run. The height of the lowest wind level in the model is 10 m in the 40 km and 12 km runs and 2.5 m for the 4 km. A detailed description of the model configuration is given by *Lean et al*. 2008. *Pearson et al*. 2010 also applied this configuration for UM simulations over southern West Africa.

[15] Most of the settings for the 4 km run are identical to the operational UM with 40 km and 12 km grid spacings, except that the 4 km model run uses a modified version of the original convection parameterization based on a convective available potential energy (CAPE) closure *Gregory and Rowntree*, 1990: the mass flux at cloud base is limited to allow most of the moist convection to be modeled explicitly. Therefore, the 4 km run is referred to as an explicit representation of deep convection. In order to allow the deep Saharan boundary layer, which can easily exceed heights of 5 km, to develop in the finest nests, the operational boundary layer scheme *Lock et al*., 2000 was replaced by a new 3-D Smagorinsky-based turbulence closure in the 4 km run.

### 2.2. Dust Emission Computations

[16] The emission fluxes of mineral dust are computed using an off-line version of the dust emission model by *Tegen et al*. 2002, which has been used for global- and regional-scale transport and radiative feedback simulations of desert dust *Stier et al*., 2005; *Tegen et al*., 2004, 2006; *Heinold et al*., 2007, 2008, 2011a, 2011b. The dust emission scheme (DES) parameterizes the flux of emitted mineral aerosol into the atmosphere as a function of the third power of the wind friction velocity, which is a measure of the kinematic stress on the surface. First-layer winds from the individual *Cascade* simulations and surface roughness from remote sensing for northern Africa *Laurent et al*., 2008 are used to calculate the friction velocity. The calculation is performed with the logarithmic wind law for neutral atmospheric stratification, assuming that the thermal stability is insignificant for the high wind speeds, which foster dust emission. The mobilization of dust particles occurs in sparsely or non-vegetated areas above a certain threshold of friction velocity that depends on the size of soil particles and surface roughness *Marticorena and Bergametti*, 1995.

[17] Here we use three different versions of the *Tegen et al*. 2002 scheme, details of which are summarized in Table 1:

1. [18] In the original version (hereafter referred to as T02), the soil size distribution is described by a combination of four lognormally distributed size classes (clay, silt, medium/fine, and coarse sand) with mode diameters between 2 μm and 710 μm, the proportion of which is derived from soil texture data on a 0.5° grid *Zobler*, 1986. The sandblasting efficiency depends on the soil texture ranging from 10^−7^ cm^−1^ for coarse sand and soils with a clay content larger than 45% to 10^−5^ cm^−1^ for silt and potential dust sources. It is assumed that the potential source areas are co-located with enclosed topographic depressions, such as paleolake and temporal lake beds, providing low surface roughness and large amounts of loose, fine-grained alluvial deposits *Prospero et al*., 2002.
2. [19] As an alternative approach (referred to as S07 hereafter), the prescription of potential dust sources is based on a dust-source-activation (DSA) frequency map. The map was derived from the infrared dust index product of SEVIRI observations aboard the Meteosat Second Generation (MSG) satellite by *Schepanski et al*. 2007, 2009. In S07, dust emission is calculated for grid cells, where at least two source activations were observed from 2006 to 2009. The surface roughness in those areas is set to a constant value of 0.001 cm.
3. [20] The third version (termed L08) uses a detailed soil size distribution database on a 0.25° grid from *Laurent et al*. 2008, which was derived using dry-sieving techniques to minimize the disruption of soil aggregates. Up to two different soil types are given per grid cell, which allows to account for subgrid-scale surface characteristics. The soil particle size distribution is represented by a mixture of four lognormally distributed modes (aluminosilicated silt, fine sand, coarse sand, salts) with mode diameters between 125 μm and 690 μm. The sandblasting efficiency ranges from 10^−6^ cm^−1^ for the coarse sand to about 2×10^−5^ cm^−1^ for agricultural soil. Instead of any a priori prescription of potential source regions, erodibility and actual dust emissions are only controlled by land-surface properties and near-surface wind speeds.

**Table:** Details of the Different Versions of the *Tegen et al*. 2002 Dust Emission Scheme

	T02	S07	L08
Reference	*Tegen et al*. 2002	*Schepanski et al*. 2007; 2009	*Laurent et al*. 2008; 2010]
Emission	following *Marticorena and Bergametti* 1995			
scheme			
Soil texture	from *Zobler* 1986,	uniform texture	from *Laurent et al*. 2008,
	except in preferential		up to 2 soil
	sources where uniform		types per grid cell,
	texture is applied		erodible fraction
Soil grain size	4 lognormal modes	lognormal modes per	4 lognormal modes
distribution	per soil class: clay	soil class: clay	per soil class:
	(2 μm), silt (15 μm),	(2 μm), silt (15 μm),	aluminosilicated silt
	fine sand (160 μm),	fine sand (160 μm),	(125 μm), fine sand
	coarse sand (710 μm)	coarse sand (710 μm)	(210 μm), coarse sand
			(690 μm), salts
			(520 μm) *Laurent et al*., 2008
Land use	27 vegetation types from equilibrium terrestrial biogeography model BIOME4 of *Kaplan* [2001]			
Preferential	enclosed topographic	dust source activation	no prescription
dust sources	depressions from	(DSA) record	required
	hydrological model	(2005–2009) from	
	HYDRA *Coe*, 1998]	MSG/SEVIRI IR	
		dust retrieval	
		*Schepanski et al*., 2007; 2012	
Sandblasting efficiency	1.0× 10^−7^– 1.0× 10^−5^ cm^−1^	1.0× 10^−5^ cm^−1^	1.0× 10^−6^– 2.0× 10^−5^ cm^−1^
Surface	derived from	derived from	updated data from
roughness	POLDER 1 satellite	POLDER 1 satellite	POLDER 1 satellite
	data *Marticorena et al*., 2004]	data *Marticorena et al*., 2004]	retrievals and
			geomorphology *Laurent et al*., 2008
Dust size distribution	8 lognormal size bins with mode radii from 0.1 μm to 1000 μm			

T02: original model by *Tegen et al*. 2002; S07: version using dust-source-activation observations from *Schepanski et al*. 2007, 2009; and L08: version based on detailed soil data from Laurent et al. (2008).

[21] Soil moisture can limit or even prevent dust emission *Fecan et al*., 1999; *Ishizuka et al*., 2008. However, since no information on the upper layer soil moisture is available from the *Cascade* model, dry conditions were assumed for the first set of dust emission computations. Potential effects of soil moisture are studied in more detail in section 4.1. The dust model is driven with hourly instantaneous wind speeds at the first model level at 2.5 m or 10 m height depending on the horizontal grid spacing (see section 2.1). Assuming that the emission fluxes remain constant during the hour, the computations yield hourly dust emissions in g m^−2^. This approach does not allow accounting for higher frequency variability, but provides sufficient accuracy for the purpose of this study.

**Figure 2:**
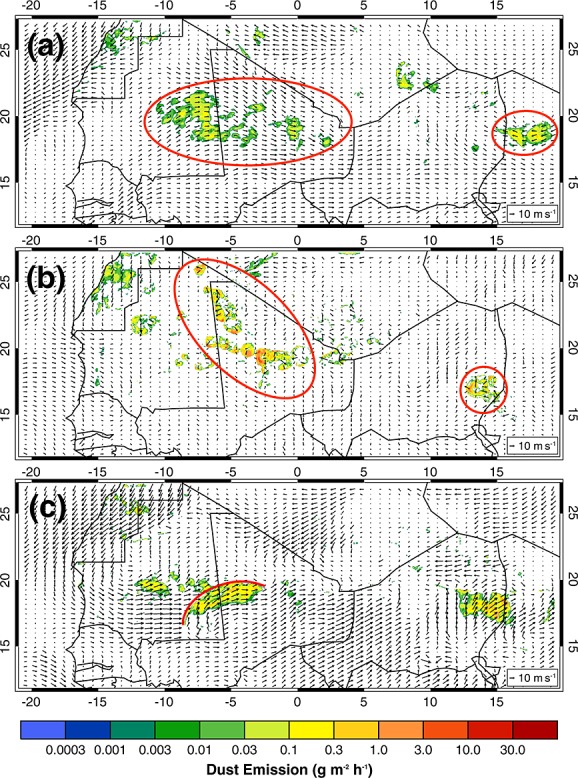
Examples of dust emission patterns indicating different meteorological drivers: (a) breakdown of nocturnal LLJs at 0900 UTC on 31 July 2006, (b) downbursts at 1800 UTC on 28 July 2006, and (c) a haboob at 0200 UTC on 03 August 2006. Instantaneous dust emission fluxes, calculated with the S07 version of DES (see section 2.2) and surface winds at 4 km grid spacing, and 925 hPa wind vectors are shown over the 4 km model domain north of 12°N. Note the logarithmic scale.

[22] The high-resolution simulations from *Cascade* show dust emission in fascinating detail. The shape and temporal evolution of dust emission patterns and the time of day of occurrence give some hints at the peak-wind-generating mechanism. Figure 2 shows examples of typical patterns found in the model results. Widespread dust emission due to the breakdown of nocturnal LLJs often occurs from Mauritania to northern Mali and in the Bodélé Depression during the morning hours (Figure 2a). Signatures of cold-pool outflow, covering many roughly circular areas of 50–200 km diameter and lasting 1–5 h, are present almost every day between early afternoon and evening (Figure 2b). These events are referred to here as downbursts. Large amounts of dust are also mobilized during the so-called haboob dust storms, caused by the large-scale outflow of organized convective systems (Figure 2c). Haboobs can span more than 500 km and are often active until the early morning hours. An animation of modeled hourly dust emissions at 4 km grid spacing is provided in the auxiliary material (Animation S1) and illustrates the modeled temporal and spatial evolution of dust uplift over the Sahel and Sahara.

[23] Many values presented in this study are area- or time-integrated dust emissions and are referred to as mean hourly dust emissions (in Mt h^−1^), dust emissions per day (in Mt), or total dust emissions (in g m^−2^). The results are presented in local time (LT) instead of universal time (UTC), since the NLLJ breakdown and the onset of convection depend on the position of the sun. LT is approximated as UTC −1 h to the west of 7.5°W, UTC +1 h to the east of 7.5°E, and UTC ±0 h in between.

### 2.3. Process Detection

[24] The cold outflow from moist convective systems and the breakdown of nocturnal LLJs are key meteorological drivers of dust emission over summertime West Africa. The occurrence of nocturnal LLJs is detected in the *Cascade* 4 km resolution run using the automated algorithm developed by *Fiedler et al*. 2013. The algorithm detects wind speed maxima below 1500 m height situated above a stably stratified surface layer of at least 100 m depth. A vertical gradient of virtual potential temperature larger than 0.1 K/100 m is used as an indicator for stable stratification. In order to limit the detection to well-defined low level maxima, a threshold of vertical wind shear of −0.5 m s^−1^/100 m is required in the first 500 m above the jet core. As NLLJs may persist after turbulent mixing has started in the morning hours, the detection algorithm also considers the so-called “NLLJ survivors”. These are vertical wind speed maxima following the above criteria but without a stably stratified surface layer. They can occur up to 3 h after identified NLLJ or “NLLJ survivor” *Fiedler et al*., 2013. In addition, we assume that it takes about 2 h for the NLLJ momentum to be completely dissipated at the surface. Therefore, dust emission events are still assigned to the NLLJ phenomenon when occurring 2 h after a detected NLLJ or “NLLJ survivor”.

[25] The 0900 LT maximum in dust emission is known to be related to the NLLJ breakdown, and the algorithm successfully identifies this process. However, the method is sensitive to the chosen settings, which need to be considered when interpreting the results. For global atmospheric re-analysis data, *Fiedler et al*. 2013 estimated the uncertainties in terms of the mean number of identified NLLJs to be less than a factor of 2. Testing the sensitivity to the wind shear criterion, the mean contribution of NLLJs to dust emission increased by a factor of 2–3 due to more identified jets while the daily and annual cycles were not substantially affected [for details, see *Fiedler et al*., 2013. In this study, an increase of the threshold for the vertical gradient of virtual potential temperature from 0.1 K/100 m to 1.0 K/100 m, as tested in *Fiedler et al*. 2013, results in a reduction of NLLJ based dust emission by 20% (cf. section 5.2).

[26] Dust emissions due to convective cold pools are detected separately. The algorithm is based on the physical principle of the formation of a cold pool or density current from the evaporational cooling of convectively generated hydrometeors sedimenting into subsaturated air below the cloud base. Dust events are assumed to be cold-pool related if the column minimum of the modeled temperature increment due to latent cooling is equal or lower than −1×10^−4^ K s^−1^ for at least one grid cell within a radius of 40 km. While convective dust storms can usually spread over several hundred kilometers *Knippertz and Todd*, 2012, and references therein], a relatively small radius is chosen here to avoid too many spurious detections. Forty kilometers is the approximate distance a cold pool can propagate within 1 h (Figure 2c), the time increment of the meteorological model output. In the same grid column, where the latent cooling criterion is satisfied, the divergence at the 925 hPa level and the maximum vertical wind speed within the 20 lowest model layers (below 2600 m) are required to exceed thresholds of 1×10^−5^ s^−1^ and 0.8 m s^−1^, respectively. This combination of criteria is very strict and targets a particular and short-lived state in the cold-pool evolution. While cold pools spread into areas of mainly hot and dry air, they transport characteristics of their warm and moist parent environment. Therefore, a threshold for the equivalent potential temperature at 925 hPa of 330 K is used as an additional criterion.

[27] The different thresholds used in this method were found by training the algorithm in sensitivity studies. Increasing (decreasing) the latent-cooling threshold by a factor of 10 leads to an increase (reduction) in total mass and relative importance of cold-pool related dust emission of only about 2% (6%) (cf. section 5.2). The emission due to cold pools increases by 13% and 4%, respectively, when the criteria for divergence and equivalent potential temperature are omitted. Without the vertical velocity criterion, up to 70% more dust emission is attributed to convective cold pools. Many of these events, however, occur around 0900 LT and are more likely related to the NLLJ phenomenon. So this criterion clearly accounts for the fact that the convective events are characterized by strong updrafts and downdrafts in contrast to NLLJ situations, which are dominated by subgrid-scale boundary layer turbulence. The tests show that the results are surprisingly robust against the sensitivity to the chosen threshold values, which give confidence in the estimates of the role of convective cold pools. Moreover, the timing of the detected cold pools in the explicit run is also consistent with the poor representation of the cold-pool winds in the parameterized run (cf. Figure 5c) *Marsham et al*., 2011a.

## 3. The Mean Diurnal Cycle

**Figure 3:**
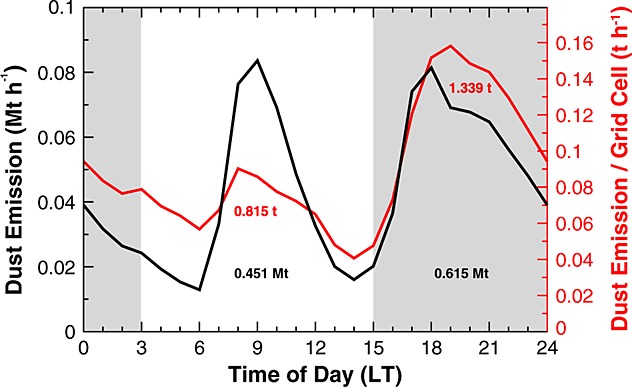
Diurnal cycle of mean hourly dust emissions (black line) and mean hourly dust emissions per active grid cell (red line) for the period 26 July to 02 September 2006. Dust emissions are computed using the S07 version of DES and surface winds at 4 km grid spacing. The color-coded numbers are total values of dust emission integrated over the periods 0300 LT–1400 LT (white box) and 1500 LT–0200 LT (grey box).

[28] The diurnal variations of dust emission averaged over the period 26 July to 02 September 2006, as calculated using the S07 version of DES and 4 km model winds, are shown in Figure 3 (black line). The mean hourly dust emissions follow a distinct diurnal cycle with a sharp maximum of about 0.085 Mt h^−1^ at 0900 LT (see section 2.2 for the definition of LT), similar to the diurnal cycle of “uplift potential” in M11. A second peak is centered around 1800 LT with about 0.077 Mt h^−1^ dust release. The smallest amount of dust particles is mobilized shortly before sunrise around 0600 LT, when the low-level stability is maximized, and during the early afternoon around 1400 LT, when boundary layer convection inhibits the synoptic flow *Parker et al*., 2005, and before the onset of moist convection. From the results, it is clear that at least three-hourly model output is needed to represent the strong diurnal cycle, while the widely used six-hourly data are less useful.

[29] Integrating the mean hourly dust emissions over the periods 0300 LT–1400 LT and 1500 LT–0200 LT yields totals of 0.451 Mt (42%) and 0.615 Mt (58%) for the morning and afternoon/evening peak, respectively. The percentages are similar to what M11 found for their “uplift potential”. M11 suggested that the morning emissions can largely be related to the breakdown of nocturnal LLJs, while the afternoon to evening emissions are largely due to moist convective cold pools (downbursts and haboobs). We will test this interpretation with the objective detection method in section 5.

[30] In addition, Figure 3 shows mean dust emission intensity (mean hourly dust emission per active grid cell; red line). Here a clear maximum from afternoon to midnight stands out with a mean hourly value of 0.154 t h^−1^ and a total of 1.339 t emitted dust per active grid cell compared to 0.064 t h^−1^ and 0.815 t, respectively, in the morning. These results show that downbursts and haboobs give more intense emissions than NLLJs and therefore have a larger impact on, e.g., air quality and transportation. This is consistent with more vigorous convective storms, but may also be due to different soil characteristics in regions dominated by cold pools or NLLJs, which are discussed later in the text.

**Figure 4:**
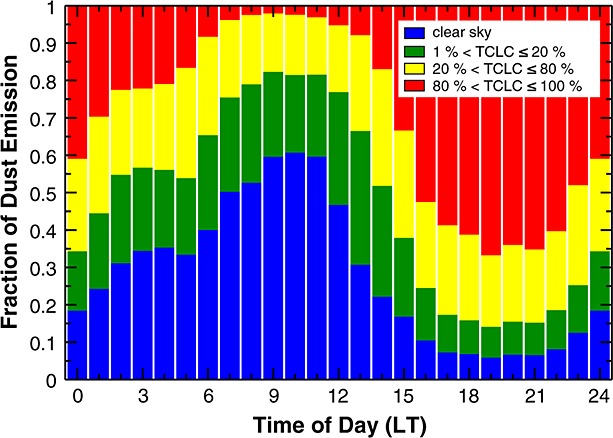
Diurnal cycle of the fraction of mean hourly dust emissions coinciding with different amounts of total cloud cover (TCLC) between 26 July and 02 September 2006. Dust emissions are calculated using the S07 version of DES and 4 km surface winds.

[31] *Schepanski et al*. 2009 estimated the importance of different meteorological processes to Saharan dust emission based on MSG SEVIRI satellite observations for the period March 2006 to February 2008. According to their results, 65% of dust source activations in the Sahara desert occur during morning hours, suggesting that the breakdown of the nocturnal LLJ is the dominant dust-generating mechanism. However, this number should be directly compared with dust fluxes, as the satellite-based source activation data only contain information on the frequency of dust events. A general issue of dust retrievals from satellite data is the lack of information under cloudy conditions. The fraction of mean hourly dust emissions covered by clouds as simulated by the model is shown in Figure 4. The cloud cover is at a minimum between 0900 and 1200 LT with up to 60% of dust being emitted under clear-sky conditions. The fraction of emissions with 80 to 100% cloud cover at this time of day is below 5%. This is exactly the time of the morning peak in Figure 3, when dust events tend to be related to the breakdown of NLLJs (M11). In contrast, up to 90% of dust emission between 1700 and 2200 LT is at least partly covered by clouds and can hardly be detected from space. A dense cloud cover of 80 to 100% obscures more than 60% of the afternoon-to-evening dust emissions. Therefore, it can be expected that all satellite retrievals representing the diurnal cycle are strongly biased toward morning dust mobilization and NLLJ breakdown.

## 4. Sensitivity Studies

### 4.1. Soil Moisture

[32] Depending on the soil texture, soil moisture can effectively suppress the emission of mineral dust *Fecan et al*., 1999; *Ishizuka et al*., 2008. Wash out by precipitation reduces the atmospheric dust load *Reeves et al*. 2010. In order to roughly estimate potential effects of these processes, we use six-hourly precipitation, as the *Cascade* model output does not provide values of upper layer soil moisture. Using this rather crude approach, dust emissions are completely suppressed if a precipitation threshold of 5 mm is exceeded in a given grid box during the 6 h ending during the particular emission event. *Belnap et al*. 2004 found that bare desert soil surfaces can dry quickly due to the high air temperatures, and also the high winds connected with dust emission events tend to dry out the soils quickly. Precipitation events less than 3 mm often result in upper soil layers drying out within less than 30 min. In this regard, the 5 mm threshold of the six-hourly precipitation gives a conservative estimate of the effect of soil moisture. Since dust transport and deposition are not explicitly computed in this study, “wash out” refers to the immediate removal of dust particles that are scavenged by precipitation shortly after emission.

**Figure 5:**
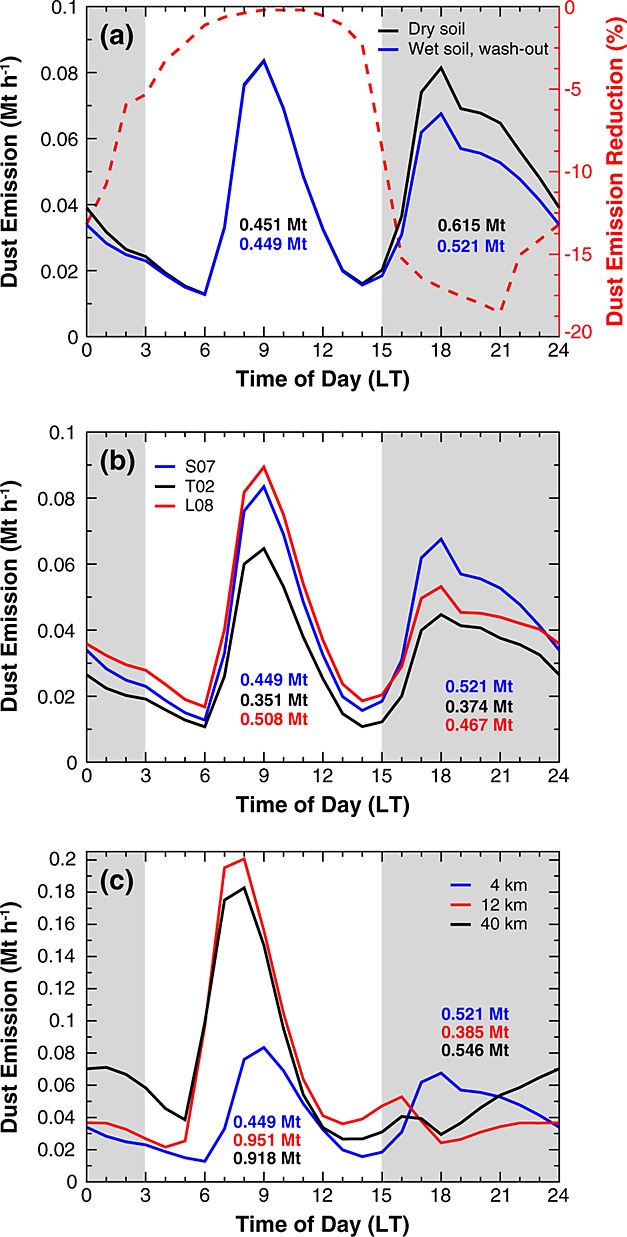
Diurnal cycle of mean hourly dust emissions for the period 26 July to 02 September 2006 as in Figure 3. Here dust emissions were computed for (a) dry and wet soil conditions (see details in the text) using the S07 version of DES and surface winds at 4 km grid spacing (the dashed red line shows the reduction of dust emission due to precipitation in percent), (b) different land-surface characteristics, i.e., DES model versions, all using winds at 4 km grid spacing, and (c) different grid spacings of the meteorological model using the S07 version of DES, integrated over the 4 km model domain. The dust emissions in Figures 5b and 5c were calculated taking the reduction through precipitation as in the blue line in Figure 5a into account.

[33] Applying this approach, the modeled total dust emission per day is reduced by 9%. While, as expected, the morning peak is hardly affected, afternoon-to-nighttime emissions are reduced by 15% due to rainfall with up to 18% reduction in the mean hourly emissions (Figure 5a). However, with 0.521 Mt, the time period from afternoon to night still contributes most to the daily dust emissions of 0.969 Mt. If we again assume a dominant contribution of downbursts and haboobs to these emissions, the results corroborate that convective outflow is a very efficient dust-mobilizing mechanism, as most cold pools apparently separate quickly enough from their parent convective storms or as a large fraction of the precipitation evaporates in the dry Saharan air.

### 4.2. Land-Surface Parameterization

[34] The dust emission calculations include different versions of land-surface parameterizations, allowing separation of effects of meteorology and surface characteristics. Taking into account the impact of soil moisture and wash out as in Figure 5a (blue line), the total amount of emitted dust per day ranges from 0.726 Mt for the original T02 dust scheme to 0.969 Mt for the S07 version and 0.974 Mt for the L08 scheme (Table 2). The diurnal cycle of dust emission is clearly dominated by meteorological factors, and not very sensitive to the surface parameterization, but the weighting varies from one DES version to the other: using the T02 and S07 schemes, a little more than half of the dust (52% and 54%, respectively) is emitted in the period from afternoon to night, while the morning emissions are slightly more important in the L08 model with a relative contribution of 52% (Figure 5b and Table 2). The reason for this is the different geographical distributions of wind speed thresholds for initial dust mobilization such that the different DES versions also favor different meteorological drivers of dust emission, dominating in one or the other region.

**Table 2:** Absolute Values and Percentages of Domain-Integrated Dust Emission Averaged Over Different Times of the Day for Different Dust Model Versions (DES, See Details in Section 2.2) Driven by Surface Winds at 4 km Horizontal Grid Spacing^a^

DES	Total Emissions	03–14 LT	15–02 LT	NLLJ	Active Cold Pool	Aged Cold Pool	Others
	per Day (Mt)	(%)	(%)	(%)	(%)	(%)	(%)
T02	0.726	48	52	46 (14)	15	22	17
S07	0.969	46	54	42 (14)	17	23	18
L08	0.974	52	48	49 (15)	13	19	19

a Relative contribution of key meteorological mechanisms as derived from the physically based detection described in section 2.3. In parentheses, the percentages of NLLJ dust emission are given, which are related to moist convection.

**Figure 6:**
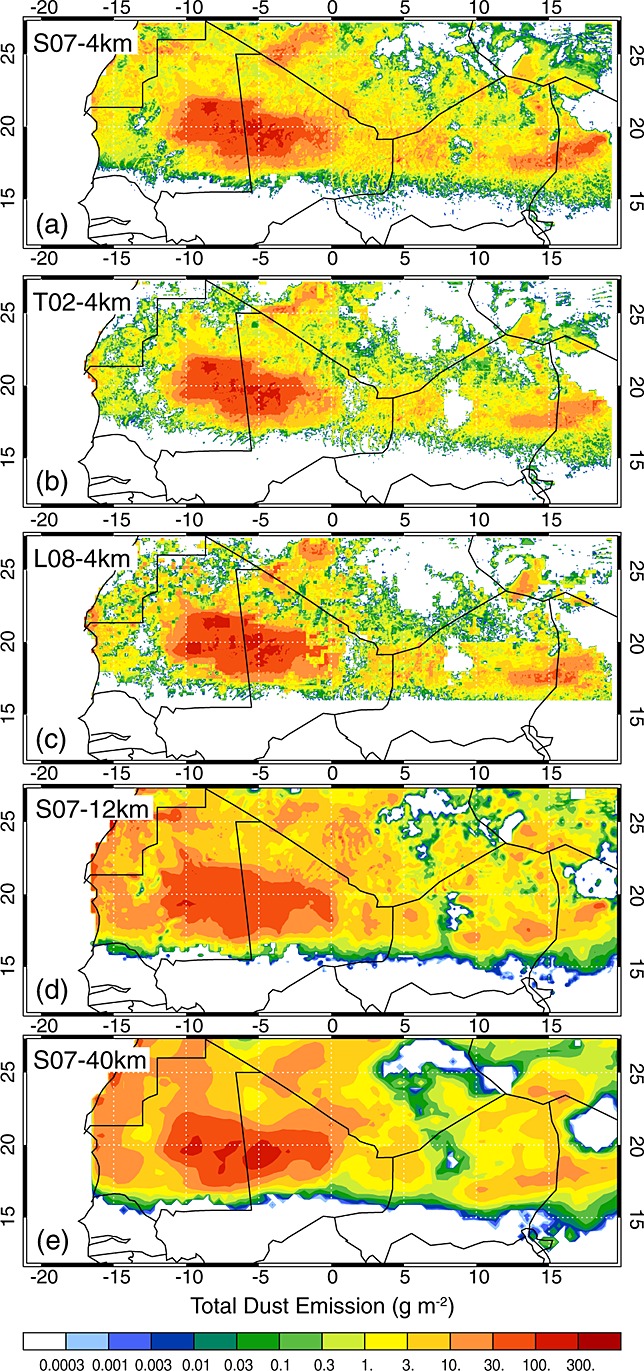
Total dust emission for the period 26 July to 02 September 2006 as computed with (a–c) different versions of DES (see details in section 2.2), all using winds at 4 km grid spacing, and (d, e) 12 km and 40 km grid spacings of the meteorological model using the S07 version of DES, shown over the 4 km domain north of 12°N (except for the L08 version, which does not extend further south than 16°N). These computations all take effects of precipitation into account as in the blue line in Figure 5a. Note the logarithmic scale.

[35] A map of total dust emissions from the three DES versions illustrates the large spatial variability of dust uplift over West Africa (Figures 6a–6c). For the period 26 July to 02 September 2006, large amounts of dust are emitted over an extensive area centered over the boarder of Mauritania and Mali, south of the Eglab Massif in Algeria, over the Bodélé Depression, and in eastern Niger. The geographical distribution is largely consistent with the “uplift potential” shown by M11, indicating that variations in wind rather than source characteristics dominate the pattern. Values of total dust emission reach up to about 140 g m^−2^ for the studied period. Differences between the schemes are found in the strength of dust emissions in hot spot areas, in particular in the source activation over the West Sahara, the foothills of the Hoggar Mountains, and the Sahel. As expected, the S07 scheme simulates the most widespread dust source activations. In contrast, the L08 version generates almost the same emission from a smaller number of activated dust sources (cf. Figures 6a and 6c).

### 4.3. Model Resolution

[36] The total amount and the diurnal cycle of modeled dust emissions depend strongly on the resolution of the meteorological simulations (Figure 5c), as shown for “uplift potential” in M11. The results of all three runs show a sharp morning peak, but considerably higher emissions are computed for the 12 km and 40 km model runs with parameterized convection. The mean hourly dust emission integrated over the period 0300 LT–1400 LT reaches values of 0.951 Mt and 0.918 Mt, respectively, which are more than twice as high as the 4 km results. On the other hand, the runs with parameterized convection show a significantly less important peak during the afternoon and evening hours with a relative contribution of only 29% (12 km) and 37% (40 km). This points toward an under-representation of cold-pool-generated dust events. A greater dust uplift around midnight partly compensates the reduction in the afternoon to nighttime emissions for the 40 km run. Interestingly, the morning and afternoon peaks occur about 1 h earlier in the parameterized runs (Figure 5c).

[37] The results indicate fundamental differences in the representation of the meteorological situation between model runs with explicit and parameterized convection. M11 found that the parameterized simulations tended to generate a deeper SHL, which leads to a stronger nocturnal acceleration of NLLJs and monsoonal southwesterlies. This can explain the much higher morning emission and the more intense uplift between 0000 LT and 0600 LT (Figure 5c). Higher wind speeds at the jet level can result in shear-driven turbulence that potentially causes an early NLLJ breakdown. However, the early morning peak may also result from the different boundary layer scheme used in the model runs with 12 km and 40 km grid spacings (see section 2.1). A comparison of the geographical distribution of dust emission shows widely similar patterns of source activation for the different horizontal resolutions, but up to 10 times higher amounts of dust are mobilized near the West African coast in the coarse-resolution runs (cf. Figures 6a, 6d, and 6e). This is consistent with an increased ventilation of the stronger SHL *Grams et al*., 2010. In this particular region, intense dust events are often simulated around 1600 LT (not shown), which is reflected in the diurnal cycle by the early afternoon peak in the 12 km and 40 km runs.

[38] In general, the sensitivity to the model resolution is much larger than the influence of precipitation and the surface scheme. While the daily (hourly) dust emission for the 12 km and 40 km parameterized runs differs from the 4 km explicit run by about 50% (factor of 2–6), a reduction of 9% (up to 18%) is found due to the effect of soil moisture, and there is a difference of 25% (up to 35%) between the T02 and S07 schemes. Most striking, however, is the dramatic change in the diurnal cycle of dust emission in the runs with parameterized convection, which is not found for soil moisture and land-surface effects.

## 5. Quantification of Key Meteorological Mechanisms

[39] For more quantitative estimates of the importance of individual drivers of West African dust release in summer, we apply the objective detection algorithms discussed in section 2.3, which help to identify whether a dust emission event is caused by the breakdown of a NLLJ or the gust front of a convective cold pool. The results show an overall detection rate of 82%. Fifty-nine percent of the emissions can be related either to a nocturnal LLJ or to a cold pool. Surprisingly, in 23% of the cases, both mechanisms are identified as drivers of dust emissions. In order to understand the circumstances of this ambiguous detection, the case of an aging cold pool is investigated in detail in section 5.1, before the mean diurnal cycle and geographical distribution of the driving meteorological processes are discussed in sections 5.2 and 5.3.

### 5.1. A Haboob Case: 26/27 July 2006

**Figure 7:**
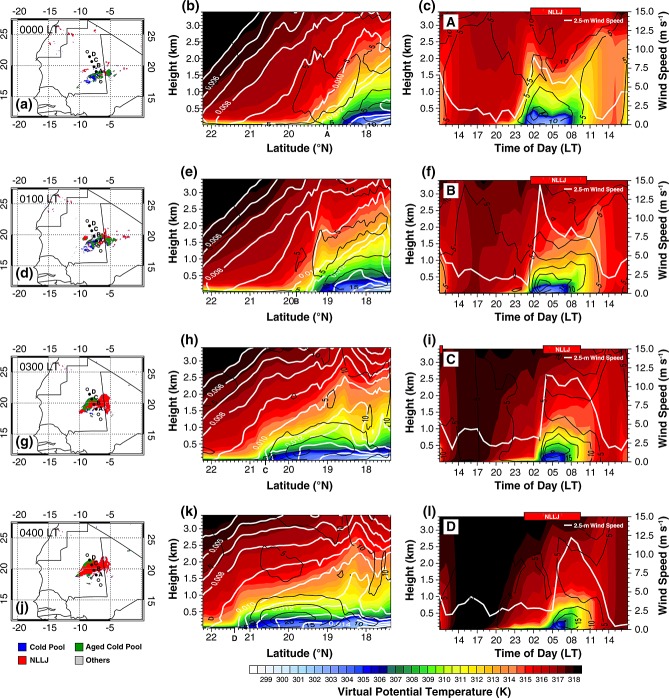
Evolution of a haboob over Mauritania on 26/27 July 2006. (left column: a, d, g, j) Hourly dust emissions colored according to the meteorological driver. (center column: b, e, h, k) Vertical cross sections through the points marked on the maps in the left column, showing virtual potential temperature (shading according to scale), specific humidity (white contours every 2 g kg^−1^), and horizontal wind speed (black contours every 5 m s^−1^). The values are zonally averaged between the easternmost (17.4°N, 6.6°W) and westernmost (22.2°N, 8.8°W) points. (right column: c, f, i, l) Time-height sections of virtual potential temperature (shading according to scale) and horizontal wind speed (black contours every 5 m s^−1^) for the sample points “A” - “D” in maps in the left column. The white lines show the first model layer wind speed at 2.5 m. The red bar at the top of each cross section indicates NLLJ detection.

[40] As an example, the haboob dust storm on 26 and 27 July 2006 is presented (Figure 7). The cold pool spreads at a speed of about 10 m s^−1^ from south-eastern Mauritania over a distance of 600 km north-westward (Figure 7, left column). Initially, the detection method indicates dust emissions due to a cold pool (blue, green in Figure 7a), but also NLLJs are found at several grid points (red, green in Figure 7a). With the movement of the cold pool, the color code changes from predominantly blue (cold pool) to green (ambiguous detection) to red (NLLJ) (Figures 7d, 7g, and 7j). An animated sequence of dust emissions split into the different driving meteorological processes (auxiliary material, Animation S2) shows the evolution of this particular cold pool and further examples in more detail.

[41] The cold pool can be clearly identified in vertical cross sections along the direction of travel of the haboob, showing virtual potential temperature, specific humidity, and horizontal wind speed (Figure 7, center column). The haboob arrives at sample point “A” around 0000 LT (Figure 7b), associated with an abrupt increase in near-surface wind speed to about 10 m s^−1^ (Figure 7c), which is well above the threshold for dust mobilization. Remarkable 15 m s^−1^ is reached at point “B” 1 h later (Figure 7f). The approach of the cold pool coincides with the onset of NLLJ detection marked by the red bar in Figure 7(right column). The cold outflow and radiative cooling of the surface lead to the formation of a stably stratified surface layer, which together with the wind speed maximum of the gust front, are exactly the conditions the NLLJ algorithm detects. This feature is clearly not a classical NLLJ resulting from boundary layer dynamics alone, but due to the changing wind structure of an aging cold pool.

[42] At a later stage, the cold pool glides up over the shallow stable nocturnal boundary layer, which has developed to the north-west ahead of the cold pool (Figures 7h and 7k). This can be hardly seen in the virtual temperature, but more clearly in the isolines of specific humidity, which are tilted backward in the beginning (Figures 7b and 7e) but later lean forward, forming a nose (Figures 7h and 7k). The cold moist air settles above the nighttime temperature inversion, where a nocturnal LLJ forms as the result of reduced frictional deceleration. The pressure gradient between the cold pool and the relatively warm residual layer can enhance the wind speed maximum aloft. As a consequence, NLLJs occur over a wide area behind the leading edge of the old cold pool (Figures 7g–7l, see also the schematic depiction in Figure 10). Shear-driven turbulence allows intermittent downward mixing of momentum from the jet level and strong dust-generating winds at night until the ultimate breakdown of the nocturnal LLJ during the morning buildup of the planetary boundary layer. The 2.5 m wind speed reflects this evolution very clearly (Figure 7, right column). A sharp peak indicates the approaching front of the cold pool, the wind speed remains high during the night, and a second maximum occurs around 0800 LT at sample points “A” and “B” (Figures 7c and 7f). A transition toward a single morning wind peak can be seen from point “C” to “D” (Figures 7i and 7l). It should be noted that dust radiative effects, which would reduce radiative cooling of the surface at night, are not considered in the model simulations. Therefore, the decoupling and NLLJ formation may be somewhat weaker in the real world.

### 5.2. Statistical Results

**Figure 8:**
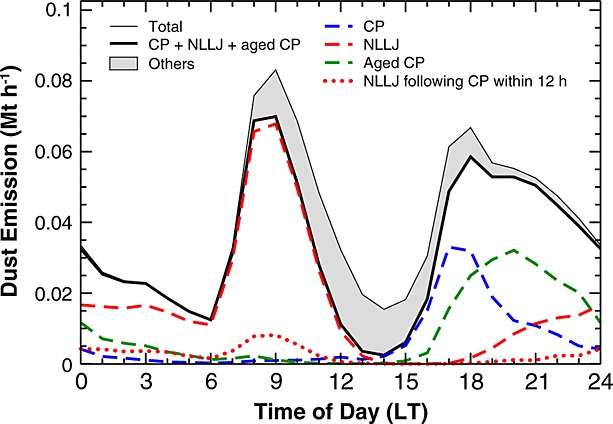
Diurnal cycle of mean hourly dust emissions for the period 26 July to 02 September 2006 as in Figure 3 (thin black line). The other solid and dashed lines show the contribution of different meteorological processes as identified using detection algorithms for convective cold pools (CP) and NLLJs. NLLJ-related emissions with a cold pool or ambiguous detection within the preceding 12 h are shown by the red dotted line. Dust emissions are calculated using the S07 version of DES and 4 km surface winds.

[43] Figure 8 shows the diurnal cycle of mean hourly dust emissions as in Figure 5a (blue line) split into the different meteorological drivers. The NLLJ-related dust emissions are characterized by a sharp rise after sunrise. Up to 75% of the morning dust uplift and 85% of the emissions at the peak time around 0900 LT can be attributed to the NLLJ breakdown, which supports the interpretation by M11. Dust emissions related to NLLJs increase again after sunset around 1800 LT, with the development of a stable nocturnal boundary layer and frictional decoupling of the flow about the surface inversion. Intermittent turbulence during the night transports momentum downward and thereby accelerate surface winds.

[44] The dust activity during the second half of the afternoon has a strong contribution from convective cold pools as expected. The peak is centered at 1700 LT, but less than half of the total detected dust emissions between late afternoon and night is unambiguously attributed to cold pools by our algorithm. A high fraction of grid boxes is found, where both a breakdown of a NLLJ and a cold pool are identified (green dashed line in Figure 8). There are strong parallels to the haboob case study in section 5.1, including the phase lag between the cold pools, ambiguous events, and NLLJ emission. This suggests that the ambiguous detections are in fact related to aged cold pools through the mechanism discussed above. Assuming that NLLJ cases, which follow a cold pool or ambiguous detection within a 12 h time period, are associated with moist convection, 6 to 14% of the NLLJ-related emissions between 0600 LT and 1200 LT and 9 to 26% at night are physically generated by cold pools (red dotted line in Figure 8).

[45] A substantial fraction of dust emissions during noon and early afternoon cannot be assigned to either of the two mechanisms. Both types reach their minimum at this time, which indicates either that about 6 hours after sunrise, the algorithm misses the remainders of NLLJs or that other processes like grid-scale dry convection, the sea breeze, or orographic channeling have forced the release of mineral dust during this time. In particular, the Atlantic inflow is a likely explanation, as it causes a maximum at this time in the 12 km and 40 km runs, but grid-scale dry convection may also contribute, since this is at maximum at this time. The rate of unclassified emission steadily decreases from 1500 LT reaching almost 0% by sunrise at 0600 LT. Overall, the detection results yield a relative contribution to the dust emissions of 42% by NLLJs and 40% by cold pools (haboobs and downbursts) with 17% from active cold pools and 23% from aged cold pools. However, also a fraction of 14% of the NLLJ detections is likely physically related to aged cold pools. About 18% could not be attributed to one of the three types (Table 2).

[46] Based on the diurnal cycle of the “uplift potential,” M11 inferred that cold-pool outflows and NLLJs each potentially generate on the order of half the dust uplift. The more sophisticated results of the detection method confirm the dominant contribution of NLLJs to the morning emissions and the role of cold pools. However, other meteorological mechanisms may become increasingly important toward noontime. The situation is less clear between noon and nighttime hours with both cold pools and NLLJs acting as drivers of dust mobilization.

### 5.3. Geographical Distribution

**Figure 9:**
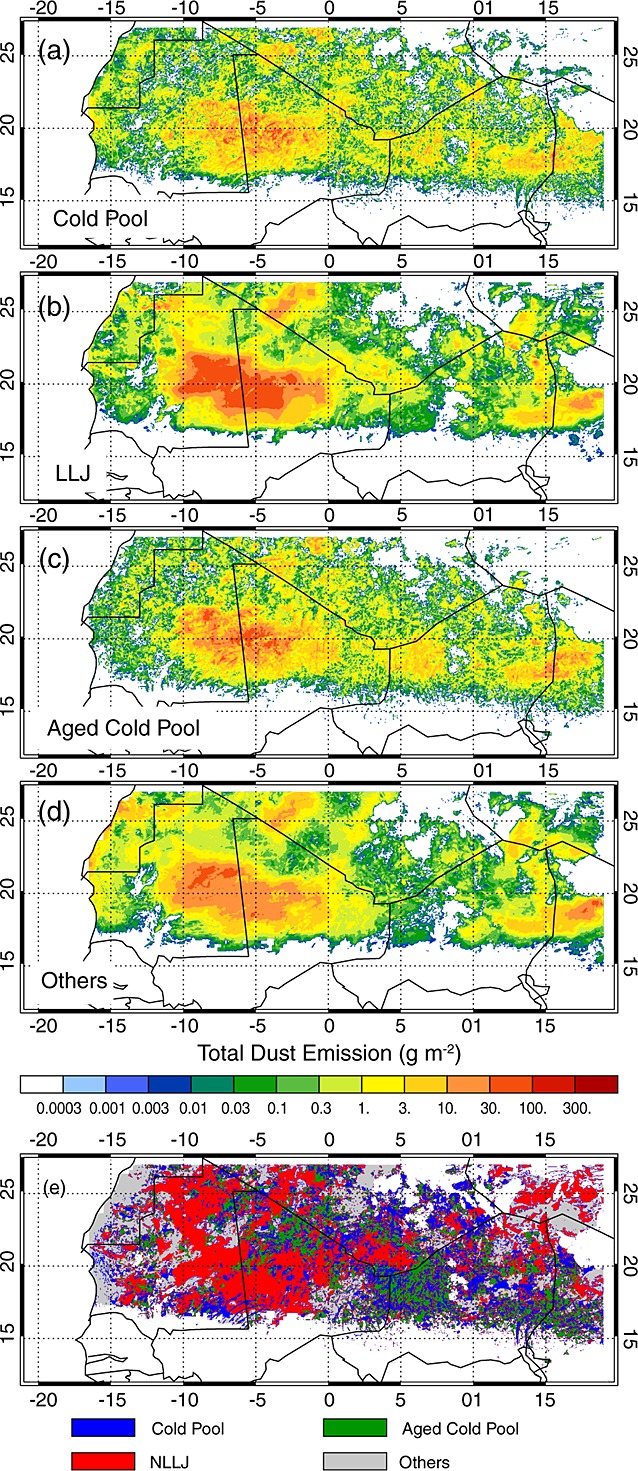
(a–d) Total dust emission for the period 26 July to 02 September 2006 as computed with the S07 version of DES and 4 km surface winds as in Figure 5a. The maps show dust emissions according to the driving meteorological processes as identified using detection algorithms for convective cold pools and nocturnal LLJs as in Figure 8. Note the logarithmic scale. (e) Map of dominant meteorological mechanisms for a given grid cell.

[47] The overall spatial distribution of dust emission is similar regardless of the mechanism (Figures 9a–9d). This indicates that within the 40 day period different kinds of peak-wind-generating processes can result in dust mobilization almost everywhere in the Sahara and Sahel. However, there are some important differences: Dust events due to cold pools show very noisy patterns because of the intense dynamics and movement of the downbursts (Figure 9a) These also occur south of 15°N. In contrast, dust emissions related to the breakdown of the nocturnal LLJ show a smooth distribution, as NLLJs are usually stationary and driven by the background pressure gradient. Dust is only emitted north of 16°N, but an additional local maximum exists between the Hoggar and Tibesti Mountains (Figure 9b). In reality, cold pools generate mineral dust in the Sahel almost as far south as 13°N *Marsham et al*., 2008; *Williams et al*., 2009, but the model does not capture these events.

[48] The synthesis of the detection results in Figure 9e shows the locations where the different meteorological drivers dominate. Haboobs and downbursts mainly mobilize dust in the eastern and central Sahel in eastern Mali, Niger, and Chad, as well as inside the Sahara in northern Mali, western Mauritania, and southern Algeria. Within 1° of Bordj Badji Mokhtar (21.4°N, 0.9°E), 62% of modeled dust emission results from cold pools or aged cold pools. This is broadly consistent with the approximately 50% from cold pools and 30% from NLLJs observed there during June 2011 *Marsham et al*., 2013, given the different months and years considered. The NLLJ breakdown forces dust emissions south and south-west of the SHL in Mauritania and Mali. These jets are embedded in the Atlantic and monsoon ventilation of the West African continent *Parker et al*., 2005; *Fiedler et al*., 2013. Other hot spots of dust events associated with the NLLJ phenomenon are situated in the Bodélé Depression, between the Hoggar and Tibesti Mountains, south of the Hoggar Mountains, and south-east of the Eglab Massif. This agrees with the fact that orographic channeling and mountain slopes favor the formation of NLLJs *Holton*, 1967; *Todd et al*., 2008. Dust production assigned to aged cold pools is predominantly found in the Sahel. Blue and green areas are often intermixed in Figure 9e, which supports a close relationship as inferred in section 5.1. The unspecified dust events occur in a belt along the West African coast and near the Hoggar and Tibesti Mountains (Figure 9d). The former suggests that the land-sea circulation and/or deflected trade winds as part of the Atlantic inflow to the SHL *Grams et al*., 2010 can be the reason for the dust uplift, while the latter points toward mountain flows as dust-generating mechanism *Birch et al*., 2012.

## 6. Summary and Conclusions

[49] West Africa is a major source of mineral dust, an important contributor to the atmospheric aerosol burden with a wide variety of impacts on the Earth System. Convective cold pools and the breakdown of nocturnal low-level jets (NLLJs) have been identified as the key meteorological drivers of mineral dust emission over West Africa during summer, but quantifying their relative contribution to the dust emissions through observations and modeling is still challenging. A unique set of convection-permitting simulations (4 km grid spacing), which were conducted with the UK Met Office Unified Model, was used here for off-line dust emission computations for a 40 day period from 25 July to 02 September 2006.

[50] The diurnal cycle of modeled dust emission shows characteristic morning peaks related to the downward mixing of momentum from nocturnal LLJs, while large haboob dust storms and small-scale downburst events typically dominate during the afternoon and night. Fifty-four percent of the dust emission occurs between 1500 LT and 0200 LT (with 49% between 1800 and 0600 UTC, comparable with the 50% observed at the Bordj Badji Mokhtar supersite-1 during the Fennec campaign in southern Algeria during June 2011, *Marsham et al*. 2013). These results are consistent with the findings by M11, who analyzed the diurnal cycle of the soil-independent parameter “uplift potential” for a 10 day subperiod. However, *Schepanski et al*. 2009 found morning emission to dominate with 65% in the Sahara based on MSG SEVIRI satellite observations for the period from 2006 to 2008, including winter months. This value refers to dust source activation frequencies, not to emitted dust mass. Keeping in mind that our study covers the 40 day period 25 July to 02 September 2006 only, it offers two explanations for this discrepancy: (1) afternoon events are more intense and (2) most afternoon dust uplift occurs under clouds. The simulations show that up to 90% (60%) of the dust mobilized from afternoon to night are partly (densely) covered by clouds, while up to 60% of the dust emissions between morning and noon occur under clear-sky conditions (consistent with the diurnal cycle in emission and cloudiness shown in *Marsham et al*. 2013). Satellite retrievals are thus expected to be strongly biased towards morning dust emissions. It should, however, be noted that the modeling of dust emission and clouds also comes with potentially large uncertainties.

[51] The dependency of the results on the effects of soil moisture and precipitation, land-surface properties, and horizontal resolution of the driving meteorological model was also investigated. Using six-hourly precipitation as a rough and conservative proxy for soil moisture and immediate wet removal, a reduction of 9% and 18% was found for daily and afternoon to nighttime emissions, respectively, while morning emissions are hardly effected. This shows that cold pools indeed are effective dust generators on average, despite the sometimes heavy precipitation of the parent convective storms. This suggests that cold pools either separate quickly from the rainfall area or form in dry regions, where evaporation rates are high and only a small fraction of the rain reaches the ground.

[52] The daily dust emission for the three different dust model versions used in this study ranges from 0.726 Mt to 0.974 Mt with similar diurnal cycles. Using detailed up-to-date soil data from *Laurent et al*. 2008 instead of the standard setup (S07) yields a slightly larger contribution from morning emissions of 52%. Each dust model has a different geographical distribution of dust sources and different threshold velocities for dust uplift. Together with preferential regions of a particular peak-wind-generating mechanism, this explains the mild differences in diurnal cycle.

[53] Much more fundamental differences are found between model runs with different grid spacings. These are most sensitive to whether moist convection is parameterized or explicitly represented. In accordance with M11, simulations with parameterized convection were found to overcompensate the dramatically underestimated moist convective activity from afternoon to night by more intense NLLJ-related dust emissions and by stronger emissions along the West African coast during afternoon as a result of a deeper SHL. Those model runs show more than twice as high dust emissions integrated over the morning hours as compared to the explicit run. Afternoon-to-nighttime emissions contribute only 30–40% to the daily dust production. In addition, the morning peak is simulated to occur earlier, which is likely due to a different turbulence scheme in the coarse-resolution runs.

**Figure 10:**
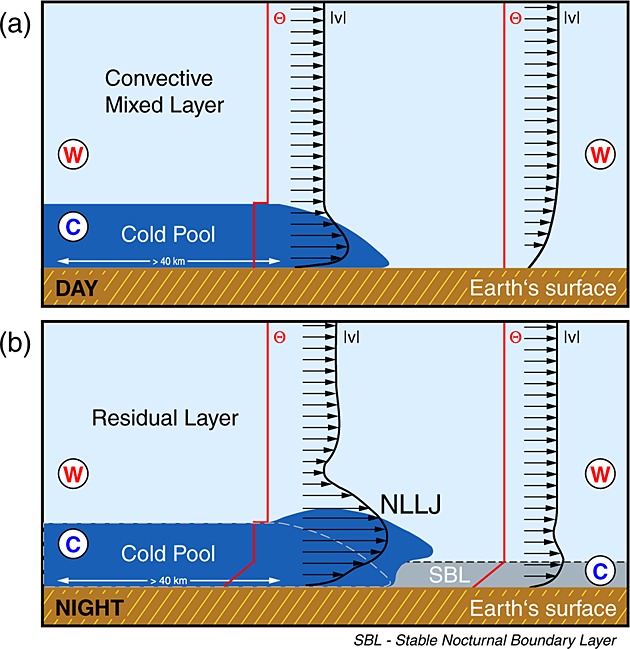
Schematic diagram illustrating the mechanism of nocturnal LLJ formation triggered by an aged convective cold pool. Shown is the (a) daytime and (b) nighttime boundary layer with typical profiles of mean potential temperature (red line) and wind speed (black line) for the undisturbed case, and the boundary layer disturbed by the cold-pool outflow. The red “W” and blue “C” indicate relatively warm and cold air, respectively. SBL stands for stable nocturnal boundary layer. For a more detailed discussion, see section 5.2.

[54] Physically based objective algorithms were applied to the high-resolution model results to detect cold-pool and NLLJ-generated dust events for a quantification of their relative importance. More than half of the daily dust emissions could be assigned to either the breakdown of a nocturnal LLJ or a convective cold pool. Surprisingly, for a considerable fraction of 23% of dust emissions both types were detected. In order to understand the circumstances of this ambiguous detection, one such case was investigated in detail, and a new mechanism was found, which is summarized in a schematic in Figure 10. The main point is that aging cold pools can glide up over a radiatively formed stable nocturnal boundary layer and trigger NLLJ formation over a wide area by locally induced pressure gradients. The spatial and temporal evolution point toward a systematic phenomenon, so that the majority of the ambiguous and some of the NLLJ emissions are physically generated by aged cold pools. The schematic in Figure 10 is consistent with observations of a real haboob case at Bordj Badji Mokhtar/Algeria in summer 2011 showing similar characteristics *Marsham et al*., 2013 and observations from the U.S. of MCS downdrafts, failing to penetrate low-level stable layers *Marsham et al*., 2011b.

[55] In summary, for the 40 day study period, the automated detection yields (1) a contribution of 42% to the dust mass emission in summertime West African by the breakdown of the nocturnal LLJ. NLLJ-driven dust mobilization dominates at the southern edge of the SHL, in the Bodélé Depression, and in mountain foothills. (2) Seventeen percent of the dust is emitted by convective cold pools in the immediate vicinity of convective storms (<40 km). They occur mainly in the Sahel region, but also inside the Sahara. (3) Aged cold pools, i.e., ambiguous detections, account for 23% of the dust emissions and an additional 14% of the NLLJ-related emissions. These often occur near regions dominated by active cold pools. (4) About 18% of the dust emissions could not be attributed to one of the three mechanisms. This fraction dominates around the midday dust emission minimum, which strongly suggests a relation to resolved dry convection, but potentially also to land-sea circulation and mountain flows, as many undetected events occur along the West African coast and near the Hoggar and Tibesti Mountains. These results support the findings by M11 regarding the role of the NLLJ breakdown, but add exciting new detail to the complex situation during the afternoon and night. These percentages are broadly consistent with the approximately 30% from NLLJ breakdown and 50% from cold pools observed at the Fennec supersite 1 in southern Algeria during June 2011 *Marsham et al*., 2013.

[56] Limitations of this study are largely related to the uncertainties of erodibility data (i.e., surface roughness and soil texture) and the physical parameterizations used to model near-surface winds and clouds. Because of the lack of systematic observations in the Sahara, we have not quantified the errors in the modeled near-surface winds relative to observations, and significant model biases may exist. Previous studies have, however, demonstrated the more realistic evolution of convection in the 4 km *Cascade* simulations compared with those with parameterized convection *Pearson et al*., 2010, K. J. Pearson et al., Modelling the diurnal cycle of tropical convection across the “Grey Zone,” submitted to *Quarterly Journal of The Royal Meteorological Society*, 2013]. There is a spatial co-location between particular uplift mechanisms and surface types, e.g., moist convection tends to form over mountains [e.g., *Birch et al*., 2012, where the land surface does not allow significant dust generation. Although we have used three surface erodibility maps in our sensitivity analysis, significant errors may remain if these maps do not capture the effects of these changes in surface type adequately. However, convection can also organize and propagate away from the mountains in the run with 4 km grid spacing. In addition, uncertainties exist due to the sensitivity of the automated detection methods to the choice of the threshold values.

[57] Continental-scale, convection-permitting simulations are computationally costly and therefore limited to relatively short time periods. This study covers a 40 day period from late July to early September of one particular year. Thus, the *Cascade* model runs do not enable to study the relative importance of the processes on a seasonal and interannual basis. Future work should investigate other cases in different seasons and years to assess the annual cycle and interannual variability of the dust-generating processes.

[58] The importance of convective cold pools and NLLJs for West African dust uplift found in this study implies further need for improvements in the representation of moist convection and stable nighttime conditions in global and regional dust models. These will allow assessing more general impacts in the atmospheric cycle of mineral dust and its environmental and climate effects.
