# Supplementary figures and images for: The role of deep convection and nocturnal low-level jets for dust emission in summertime West Africa: Estimates from convection-permitting simulations
Source: J Geophys Res Atmos. 2013 May 29;118(10):4385–400. doi: 10.1002/jgrd.50402 (PMC4394720; doi:10.1002/jgrd.50402)

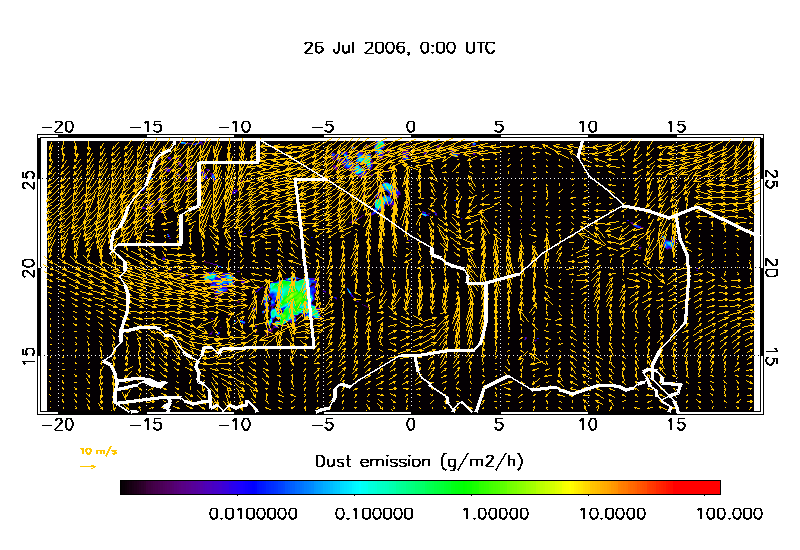

Supplement: Supplementary file 1 [file jgrd0118-4385-SD1.gif]

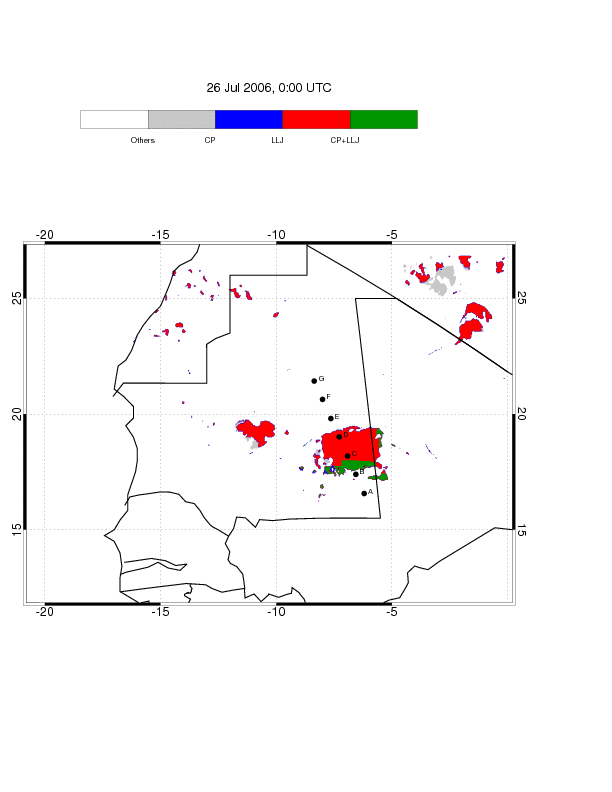

Supplement: Supplementary file 2 [file jgrd0118-4385-SD2.gif]
